# Auricular Neuromodulation: The Emerging Concept beyond the Stimulation of Vagus and Trigeminal Nerves

**DOI:** 10.3390/medicines5010010

**Published:** 2018-01-21

**Authors:** Beniamina Mercante, Franca Deriu, Claire-Marie Rangon

**Affiliations:** 1 Department of Biomedical Sciences, University of Sassari, Sassari 07100, Italy; bmercante@uniss.it; 2 Head of Scientific Auriculotherapy Diploma, Faculty of Medicine, University of Paris-Saclay, Saclay 94270, France; cmrangon@gmail.com

**Keywords:** neuromodulation, vagus nerve, trigeminal nerve, auricle, auriculotherapy, auricular acupoint, peripheral neuromodulation

## Abstract

Neuromodulation, thanks to intrinsic and extrinsic brain feedback loops, seems to be the best way to exploit brain plasticity for therapeutic purposes. In the past years, there has been tremendous advances in the field of non-pharmacological modulation of brain activity. This review of different neurostimulation techniques will focus on sites and mechanisms of both transcutaneous vagus and trigeminal nerve stimulation. These methods are scientifically validated non-invasive bottom-up brain modulation techniques, easily implemented from the outer ear. In the light of this, auricles could transpire to be the most affordable target for non-invasive manipulation of central nervous system functions.

## 1. Neuromodulation and Neurostimulation

Neuromodulation is a physiological process which consists of the alteration of neuronal and synaptic properties by neurons or substances released by neurons [1]. 

In recent years, the use of electrical- and/or magnetic-based neurostimulation methods has raised a growing interest both in clinical and experimental research. Although our scientific knowledge is still incomplete, a relevant amount of evidence allows us to affirm that whatever the stimulus used, with both central and peripheral approaches [2,3], neurostimulation impacts on the release of neurotransmitters and thus on the activity of the central nervous system (CNS). By changing neurotransmitters’ concentration in specific brain regions, neuromodulation may modify the intrinsic properties of neuron membrane thus altering response to synaptic events. Indeed, neuromodulation does not necessarily convey excitatory or inhibitory signals, but rather alters the cellular and/or synaptic properties of neurons [4]. As a consequence, neuromodulatory stimuli may influence the development of new neural circuits or reshape the output of the existing ones, sometimes effectively rewiring or reprogramming them [5,6,7]. In this light, neuromodulation should be seen as a manipulation of the balance between different functional brain networks, which can result in improved behaviour when applied in the right context [8]. 

Some neuromodulatory substances have been termed ‘extrinsic’ as they are released by neural projections that are clearly outside of, or not part of, the circuits that they modulate [9,10]. Others are termed ‘intrinsic’ because they are released by some of the self-same neurons that are part of the circuit that they modulate [8,9,10]. In this case, when the circuit is operational or active, some of its neurons may release neuromodulators that alter synaptic strengths and intrinsic membrane properties of other circuit components [6,10].

Extrinsic modulation can be used not only to regulate one neural circuit, but also to organize ensembles of circuits found in myriad regions in the CNS. Intrinsic modulation may be more restricted, both spatially and temporally, as it may be used primarily to maintain ongoing activity. For example, in a circuit with significant intrinsic modulation that produces an enhanced level of excitability, a short synaptic input can ‘jump start’ a circuit, causing the release of an intrinsic modulator that would significantly maintain the activity after the initiating signal [10]. Because it is now clear that many neurons may have both ionotropic and metabotropic receptors to the same neurotransmitters, it is possible that some amount of metabotropic-mediated intrinsic modulation commonly accompanies strong circuit activation that releases a significant amount of transmitter [6,7]. As discussed above, neurostimulation is one method used to modulate the information processing activity of the CNS. This is typically carried out to compensate for loss of normal function due to disease or injury. Implicit in the use of neurostimulation to treat a neural disorder is a rudimentary understanding of which part of the nervous system is dysfunctional and the type of compensation required to recover normal function [11].

However, not all neurostimulation approaches end up with modulation of CNS activity or neuronal plasticity. Indeed, some consequences of neurostimulation are most likely due to activation of nervous reflex circuitries rather than to neuromodulation [12]. A prototypical example is the inflammatory reflex, comprised of an afferent arm that senses inflammation and an efferent arm, the cholinergic anti-inflammatory pathway, that inhibits innate immune responses and the progression of inflammatory diseases [12,13]. The inflammatory-reflex is an example for how action potentials of neurons belonging to integrated neural network impact on immunity. In addition, in this case a fundamental role is played by neurotransmitters which propagate essential information for the regulation of immune responses, steering together the fields of immunology and neuroscience [13,14]. Tonic neural activity in the cholinergic anti-inflammatory pathway is essential for health because when it is impaired the consequences include unrestrained cytokine responses that damage tissue or kill the host. By contrast, enhancing the activity of this immune-mediated neural circuit confers protection [14]. In this perspective, neurostimulation methods have been developed to target neural networks for the treatment of inflammatory disorders.

Neurostimulation provides a much-needed therapeutic relief for an extraordinary number of people affected by debilitating neurologic and psychiatric disorders worldwide.

From a medical viewpoint, neurostimulation techniques do provide several advantages with respect to conventional drug treatment:

- Specificity: stimulation can be targeted to particular areas avoiding the insurgence of systemic side-effects, typical of traditional drug therapies;
- Safety: neurostimulation techniques are generally well-tolerated and almost devoid of dangerous side effects;
- Flexibility: the treatment can be interrupted at any time.

In its most basic form, a neurostimulation device is constituted by: a power supply, a programmable pulse generator, the electrodes in contact with the tissue and the connection wires. Stimulation parameters (waveform, amplitude, frequency, pulse duration and duty cycle) and system placement depends on the device type, the anatomical location of the targeted dysfunctional neuronal circuitry and the patient’s medical history [15].

Based on the anatomical placement of the stimulation device, neurostimulation approaches may be classified as invasive and non-invasive. 

Invasive neurostimulation therapies have emerged as an effective treatment for a growing number of medically resistant neurologic and neuropsychiatric disorders. However, their use is hampered by the cost and the need of surgical implantation which is not devoid of risks and side effects, hence different non-invasive techniques have been evaluated and increasingly developed as alternative approaches.

Non-invasive neurostimulation include several techniques largely used as a treatment for many neurologic and neuropsychiatric disorders. Among them, transcutaneous electrical nervous stimulation (TENS) has shown a rapid evolution in the last decades. The low cost, portability and ease of use are among the many characteristics that made TENS techniques very interesting to the scientific and medical communities. Indeed, their simplicity and versatility are an attractive option for the clinician as a stand-alone therapy or combined with other neuromodulation treatments [3]. Among the different TENS-derived techniques, a large body of evidence now demonstrates that both transcutaneous trigeminal and vagus nerve stimulation (TNS and tVNS, respectively) can exert robust therapeutic effects without unsafe consequences.

## 2. TNS, tVNS and Auricular Stimulation: Possible Sites and Mechanisms of Action in the Central Nervous System

**TNS.** An increasing bulk of literature is giving evidence on consistent beneficial effects exerted by TNS on the symptoms of drug-resistant epilepsy [16,17,18,19,20,21,22], migraine [23,24,25,26], and depression [2,17,27,28,29]. From a clinical and experimental point of view, TNS produces similar effects to the well acknowledged invasive vagus nerve stimulation (VNS) but without its autonomic side effects. Moreover, TNS can be delivered bilaterally at low stimulation intensities, with potentially greater beneficial effects with respect to VNS [30]. Indeed, TNS side effects are uncommon, temporary and mild and include skin irritation, tingling, forehead pressure, and headache [28,30]. TNS has been proved to be safe, not associated with adverse cardiovascular events and to have some advantages in comparison with VNS [31]. TNS is commonly delivered by applying the stimulating electrodes on the skin over the emergence of the supraorbital and/or infraorbital branches of the trigeminal nerve [21]. 

It is interesting to note that a decrease in vigilance and arousal after or during TNS administration has been incidentally reported by several studies [32,33] and demonstrated by psychometric testing in healthy subjects [34]. It has been shown that this effect is not attributable to a decrease in sleep latency, namely to an hypnotic effect [34,35]. Although the nature and the extent of this phenomenon is still unclear, it has been proposed that it may be attributable to the influence of TNS on the activity of monoaminergic brainstem nuclei, such as the locus coeruleus. Indeed, if confirmed, such an effect may give rise to interesting perspectives for a drug-free approach of hyperarousal states and, possibly, anxiety-derived sleep disorders. However, more in-depth investigation possibly performed with adequate methodology (e.g., quantitative EEG analysis and psychophysical tests), is definitely needed.

A recent immunohistochemical study in rats has demonstrated that TNS induces a c-Fos expression not only in the trigeminal sensory nuclei, thalamus and somatosensory cortex, but also in the nucleus of the solitary tract, the locus coeruleus and the dorsal raphe nucleus bilaterally [36]. In addition to these brainstem regions, a significantly c-Fos labelling was found in forebrain structures such as the endopiriform nucleus, the entorhinal cortex, the hippocampus and the amygdala [36]. These data provide the anatomical support to physiological results proving that TNS affects brainstem structures [32,33,35] which in turn influence forebrain areas involved in the pathophysiology of specific CNS disorders, such as epilepsy and depression, on which TNS has been shown to exert therapeutic effects [17,19,26,31]. In this sense, particularly interesting are recent findings proving that short-term TNS induces a significant cellular proliferation in the dentate gyrus of the hippocampus [36] and chronic TNS reduces hippocampal apoptosis described in epileptic rats [37]. In line with these data are the results of a behavioural study in a rat model of experimental epilepsy which showed that acute TNS is able to modulate seizure type towards a decreased duration of the most severe tonico-clonic seizures [36]. 

Interestingly, short-term TNS has been shown to exert long-term inhibition on brainstem interneurons, which resembles a long-term depression effect [33]. This finding suggests that plastic changes in nervous circuits may occur following TNS, in line with those described following VNS [38]. 

**tVNS.** Compared to invasive VNS, tVNS has several advantages and in particular does not require surgical intervention for the implantation electrodes around the left branch of the vagus nerve. This procedure is not risk-free, is expensive, its long-term effects are not known and bradycardia due to unwanted stimulation of efferent fibres of the vagus nerve is always a possibility. Further, in case of dysfunctions or battery replacements, another surgical intervention is necessary. 

While tVNS can be administered at the neck level for the acute treatment of pain associated with episodic cluster headache in adult patients (gammaCore.com^®^, approved by FDA in 2017), an alternative method has been proposed theoretically by Ventureyra (2000) [39], aiming at the cutaneous representation of the vagus nerve in the external ear [40]. 

It is noteworthy that the external ear is the only place on the surface of the body with afferent vagus nerve distribution that provides, through the auricular branch of the vagus nerve (ABVN), somatic afferents from the ear to the spinal trigeminal nucleus, which, along with the principal trigeminal nucleus, is the main recipient of the trigeminal afferent system. The external ear is also innervated by trigeminal nerve fibres, travelling in the auriculotemporal nerve (ATN), which partially overlap with the vagus territory, as described in detail below.

**Auricular stimulation.** The literature on the therapeutic effects of auricular stimulation on the symptoms of several disorders is greatly increasing [41,42,43,44,45,46,47] and these studies are showing that the beneficial effects induced by VNS and TNS can be reproduced by non-invasive auricular stimulation [48,49].

**Figure 1:**
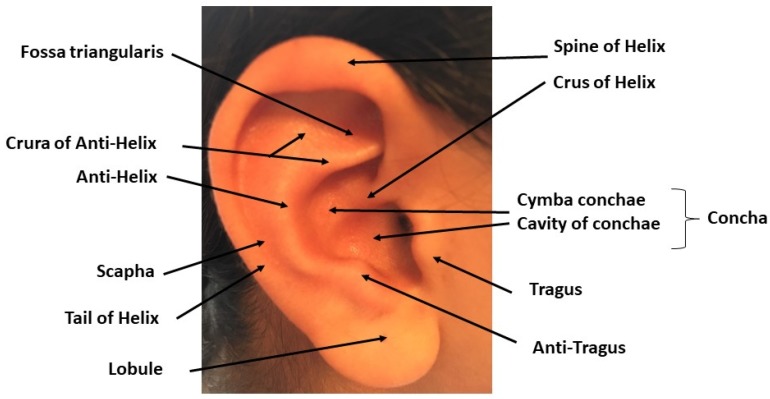
Gross anatomy of the Auricule: the main components of the outer ear are named and pointed on the picture. Copyright Dr. Rangon.

The auricle (Figure 1) consists of the helix (the folded over outside edge of the ear), the antihelix (forms a Y shape), the fossa triangularis (the depression in the fork delineated by the crura of antihelix), the scapha (the depression between the helix and the antihelix), the concha (the hollow next to the ear canal made of two parts: the upper one, the cymba conchae and the lower one, the cavity of conchae), the tragus (a small eminence in front of the concha), the antitragus (below the tragus) and the lobule (the lowest part of the ear and the only one that does not contain cartilage).

According to the study by Peuker and Filler (2002), performed on seven cadavers, cymba conchae is exclusively supplied by the ABVN; cavity of conchae is supplied by ABVN solely in 45% of cases and by both ABVN and the great auricular nerve (GAN) of the cervical plexus (C1, C2) in 55% of cases ; tail of helix and the scapha, the lobule and the antitragus are exclusively supplied by GAN; antihelix is supplied by ABVN solely in 73%, exclusively by GAN in 18% and by both ABVN and GAN in 9% ; the crura of antihelix are supplied by GAN in 91% and ABVN in 9% ; the crus of helix is supplied in 80% by the trigeminal ATN and in 20% by the ABVN; the spine of the helix in 91% by ATN and GAN in 9% ; at last, tragus is supplied exclusively by GAN in 45%; in 9% by ATN solely and by both in 47%.

**Figure 2:**
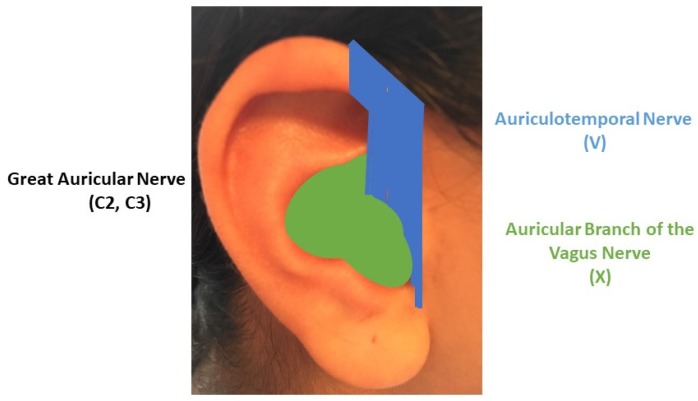
Delineation of three strategic areas for non-invasive neuromodulation on the lateral side of the auricle: the green area is mainly supplied by the Auricular Branch of the Vagus Nerve (X); the blue area is mainly supplied by the Auriculotemopral nerve, a branch of the trigeminal nerve (V); the largest remaining area is mainly supplied by the Great Auricular Nerve (C2, C3). Inspired by Peuker and Filler, 2002. Copyright Dr. Rangon.

In light of the above anatomo-physiological data, we suggest a schematic delineation of three main regions for auricular neuromodulation strategies (Figure 2) of CNS areas considered to be crucial in the physiopathology of several disorders [50]. In this sense, external ear could turn out to be one of the most accurate and powerful at hand tool for brain neuromodulation. 

The first area consists of the concha (green area) and the most cranial part of the medial side of the outer ear. This area, mostly supplied by the ABVN, is the auricular tVNS target zone. Not surprisingly, the cymba conchae, exclusively supplied by the ABVN [51] has been proposed by a recent fMRI study as the “optimal location for tVNS therapies applied to the auricle” [52]. 

At the moment, several devices are commercially available including NEMOS^®^ (www.cerbomed.com) which stimulates the cymba conchae for the treatment of epilepsy, P-stim^®^ (www.octusaspine.com/p-stim.html), which stimulates acupunctural points in the auricle for the management of chronic pain, and SaluStim^®^ (www.tinnitustreatmentcentre.com), designed to relieve tinnitus by applying tVNS at the inner side of the tragus in combination with sound therapy [52].

On the neurophysiological side, several studies have shown that far field potentials, called vagus-somatosensory evoked potentials, were recorded from the scalp following stimulation of the inner side of the tragus of the contralateral ear, at latencies compatible with a brainstem origin [53,54]. Recently, the accessibility of the central projections of the vagus nerve via electrical stimulation of the external ear has been also demonstrated by functional Magnetic Resonance (fMRI) studies [42,44,52,55]. These studies have shown that the stimulation of the cymba concha induces the activation of the spinal trigeminal nucleus and of the nucleus of the solitary tract. Other more cranial regions, previously identified as main target of invasive VNS [56] and TNS [36], such as the locus coeruleus and the parabrachialis nucleus are also activated by the stimulation of the cymba concha. At the midbrain level, the periaqueductal grey matter, the dorsal raphe nucleus, the substantia nigra and the red nucleus were the regions significantly activated by cymba concha stimulation in comparison with a control stimulation. In the forebrain, besides the primary somatosensory area, the amygdala, the fornix, the thalamus and the insula were consistently activated. Interestingly, recent data have shown that the hippocampus and the hypothalamus are also a target for auricular stimulation but the observed effect was deactivation [55]. The activation/deactivation effects observed with fMRI in specific areas of the CNS, including the hippocampus, over last the duration of the cymba concha stimulation [55] providing support to the long-lasting effects of auricular stimulation.

Another recent interesting experimental finding is that stimulation of the ABVN in the area of the auricular concha plays an important role in immunoregulation through the activation of the cholinergic anti-inflammatory pathway and the downregulation of proinflammatory cytokine expressions and nuclear factor (NF-κB) activities [43,57]. This is consistent with a previous study showing that VNS inhibits the systemic inflammatory response to endotoxin (lipopolysaccharide) administration, via the release of acetylcholine [12,13,14]. More recently, it has been found that VNS might downregulate inflammatory cytokine release, providing support for its anti-inflammatory effect [58]. It has been proposed that the stimulation of the ABVN, via tVNS, activates the nucleus of the solitary tract and that the integrated output is carried by the efferent vagus nerve to inhibit the inflammatory responses [43]. Furthermore, animal studies reported that auricular stimulation reduces infarct volume, induces angiogenesis after cerebral ischemia and may therefore represent a new potential therapeutic tool for ischemic stroke [59,60]. These data are supported by the evidence that VNS paired with rehabilitative training confers significantly improved forelimb recovery after intracerebral spontaneous hemorrhage in rats compared with rehabilitative training without VNS [61,62]. These data, obtained in experimental animals, indicate that tVNS treatment may improve recovery of neurological function [59,60,61], however human studies have yet to be undertaken. 

The second auricular neuromodulation area, composed of the lateral anterior part of both the helix and antihelix plus the tragus (blue area), is mostly supplied by the ATN. This area could be the auricular target zone for TNS. It is noteworthy that this area contains, among others, the “lateralization point”, known to facilitate general analgesia [63], or the “hunger point”, whose stimulation helps to lose weight [64]. TNS activates nervous structures in the brainstem and forebrain which have been proven by neuroimaging studies in humans to be also a target for afferent fibres innervating the external ear, namely the cymba concha [42,44,52,55]. The recent finding that electrical stimulation of the tragus can reliably activate cerebral afferent vagal networks on fMRI [65] strongly supports auricular TNS selection. 

The third and largest remaining area of the auricle, supplied by the cervical plexus, consists of the posterior part of antihelix, helix and scapha, plus the antitragus, the lobule and most of the medial side of the ear. According to auriculotherapy, this area should be the target zone to be explored for diseases involving distinct brain lesion/dysfunction and motor rehabilitation. 

**Figure 3:**
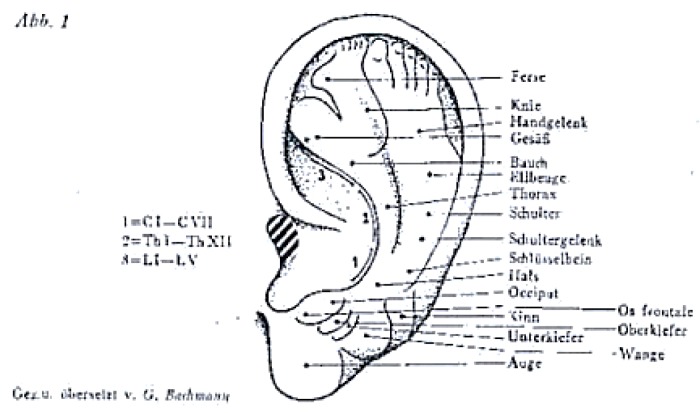
The first Auricular map published by Paul Nogier in 1957. The head is represented towards the lower lobule, the hands and feet are represented at the uppermost portion of the auricle and the body in between. Copyright Raphael Nogier. Published with his authorization.

Indeed, in 1957, the first hypothetical map (Figure 3) of different body regions appearing on the external ear as an inverted foetus [66] represented the head towards the lower lobule. 

Several later experiments supported the theory of auricular somatotopic cartography: the “thumb pinching test” [67] showed a correlation between the disappearance of a thumb pain after removing a clamp and changes in the tenderness of the auricular zone of the thumb. A decreased electrical skin resistance has also been proposed for individuating the location of ear acupuncture points [68,69] and fMRI findings supported the connection between a zone of the auricle and a particular organ through the stimulation of the same somatosensory cortex area [70,71].

Ear maps were gradually enriched with points corresponding not only to body organs but also to central nervous system structures such as thalamus, basal ganglia, anterior commissure with many differences among the various cartographies developed. The World Health Organization (WHO) contributed to standardization by developing the first International Nomenclature in 1990. 

Twenty one years later; the World Federation of Chinese Medicine Societies (WFCMS) ratified a proposed “biomathematical model of the brain organization” to which the auricles are connected [72]. Nevertheless; the representation of the CNS structures within the helix and lobule of the auricles still require scientific validation [73]. 

## 3. The “Next Frontier” of Neuromodulation of the Outer Ear: The Cervical Plexus Supply Area

It is worth noting that this particular area of the outer ear has been almost underexploited for clinical electrostimulation so far. For example, a recent study [74], points out that auricular points used for treatment of pain in 17 randomized controlled studies did not belong to this area but were exclusively located in areas of the ear innervated either by cranial nerves only (including X and V) or in areas with mixed innervation by the vagus nerve and cervical nerves. Indeed, the area innervated by the cervical nerves, mainly the lobule, is often used in experimental studies as a control site [75,76,77]. Nevertheless, ear-point electrical stimulation (0.2 mA, 80 Hz) on the great auricular nerve was able to definitely improve both electrocorticogram and seizure behavior in one study on epileptic induced rat [78]. Another controlled Chinese study assessed the therapeutic effect in vascular dementia of auricular point taping and pressing therapy of Shennen Nao (brain), Shen (kidney) and Zhen (pulvinal) [79]. This study included two groups of 90 patients treated during 12 weeks either with auricular point taping and pressing or with oral Nimodipine (3 × 30 mg per day) and showed no differences between the two groups. Only one fMRI study has supported the representation in the CNS of the ear area supplied by the cervical plexus area [80]. This study was originally designed to validate the specificity of ear acupuncture. It compared two acupoints, the Brain Stem Auricular Acupoint (BSAA, an acupoint of a CNS structure) and the Thumb Auricular Acupoint (TAA, an acupoint of a body point), located in two different parts of the ear (respectively, the antitragus and the upper part of the scapha). It gave evidence that the stimulation of BSAA, mostly activated cortical and limbic regions on fMRI whereas the stimulation of the TAA selectively activates the secondary somatosensory area bilaterally [80]. Nevertheless, this small study only involved six volunteers (three males and three females) and no scientific conclusion could be drawn. BSAA is the first of a long list of CNS auricular acupoints whose location is waiting to be validated by modern research.

According to auriculotherapy, the medial side of the auricle is thought to regulate motor and endocrine paradigms whereas its lateral side is supposed to be involved in sensorial and sensitive matters. It is noteworthy that the medial side of the outer ear is almost exclusively supplied by the cervical plexus. An experimental study, using electrostimulation of the lateral side of the ear (cathode in the apex and anode in the lobe of the ear: frequency: 2 Hz, intensity: 2 mA, duration: 100 μs; 20 min/day, 10 min for each ear for 7 days continuously) was not able to improve motor scores but significantly ameliorated learning and memory impairment in rats with ischemia-reperfusion injury [81]. The authors explained the absence of significant improvements in the motor domain with the reperfusion protocol used (ear stimulation 24 h after reperfusion) and attributed the cognitive beneficial effects with neuroprotection related to the increase in acetylcholine release and in neuronal survival [81]. 

In light of the above data, the ear area innervated exclusively by the cervical plexus (basically the lobule and the lowest part of the helix), seems to be the “next frontier” for scientists to explore and unravel, probably opening the way to brain area targeted and/or motor neuromodulation. 

## 4. Concluding Remarks

Thanks to the availability of high-tech devices and scientific progress, in less than a century since the hypothesis of auricular maps by Paul Nogier, we are about to start advanced multi-modal neuromodulation through the ear. Auricles might then become the most affordable, accurate and powerful gateway to the brain, if ever the auricular maps, used for years in auriculotherapy, are fully proven to be scientifically correct. Comparative studies evaluating the ratio efficacy/cost of auricular stimulation versus other neurostimulation modalities are warranted and would probably boost this emerging technique, not only helpful for therapeutic purposes but also for understanding healthy humans functioning [82].

## Abbreviations

ABVN	Auricular Branch of the Vagus Nerve
ATN	Auricular Temporal Nerve
BSAA	Brain Stem Auricular Acupoint
CNS	central nervous system
FDA	Food and Drug Administration
fMRI	functional Magnetic Resonance Imaging
GAN	Great Auricular Nerve
TAA	Thumb Auricular Acupoint
TENS	transcutaneous electrical nerve stimulation
TNS	trigeminal nerve stimulation
tVNS	transcutaneous Vagus Nerve Stimulation
VNS	Vagus Nerve Stimulation
WFCMS	World Federation of Chinese Medicine Societies
WHO	World Health Organization
